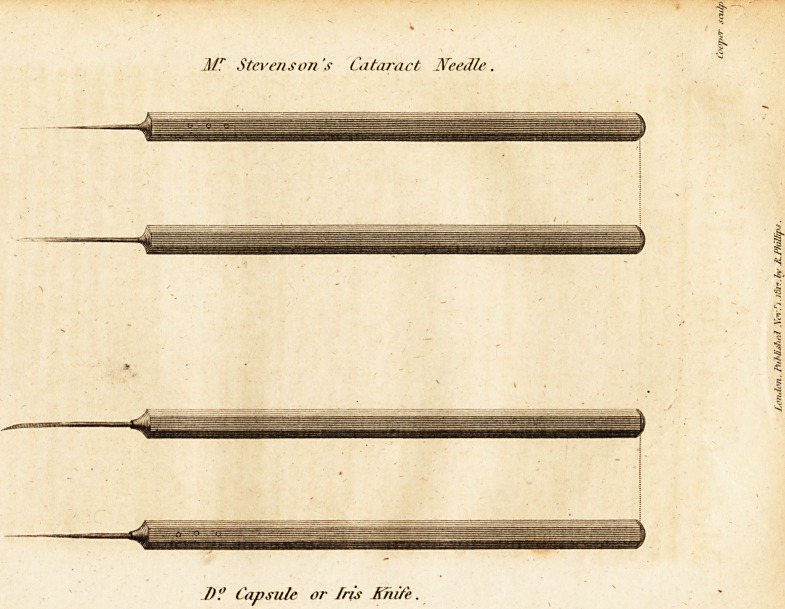# Mr. Stevenson on Cataract

**Published:** 1812-10

**Authors:** 


THE
### Medical and Physical Journal.

4 OF VOL. XXVIII.] OCTOBER, 1812. [NO. 164.

### *To the Editors of the Medical and Physical Journal.*

GENTLEMEN,

DEEPLY impressed with the disinterested conviction, that the improved method of curing the different species of cataract suggested by the late Mr. Saunders, ought not to be longer withheld from the profession at large, early last spring a paragraph was inserted in the daily newspapers, expressive of my intention of speedily and unreservedly communicating to the public the whole process, in a practical Treatise on that interesting and important subject. Whether from that or any other cause I do not pretend to surmise, yet, certain it is that, by a singular coincidence, in a very few days subsequently to that notification of my design, his long-promised posthumous work actually made its appearance in print. My wishes being by that event very unexpectedly realised, and finding myself at that time busily engaged in professional pursuits, my further labors with respect to the proposed undertaking (in which considerable progress had been made) were immediately suspended. From the perusal of the book in question, I learnt, to my inconceivable astonishment, that Mr. S. had not left any express manuscript account of his operations for cataract; a deficiency which the editor, consequently, was under the necessity of supplying.

During the period I enjoyed the privilege of Mr. Saunders's public as well as private tuition relative to his practice in diseases of the Eye and Ear, he not only afforded me, in our evening visits to his patients, opportunities of witnessing the progress of several of his cataract cases, but was likewise in the constant habit of familiarly expatiating upon the nature and comparative advantages and defects of his own operation, as contrasted with couching and extraction; the latter of which, under certain circumstances, he even then thought preferable. Not content with these proofs of friendship, and with verbal discussions on the above points, when I finally took leave of him he voluntarily tendered a promise to favor me with a written document explanatory of the method he pursued in the operation. That he hastened to redeem the pledge he had so obligingly given to me, the following extracts from his confidential letters, will abundantly evince. The publication already alluded to, having wholly released me from any breach of honor in making their contents known, it will, I trust, be deemed highly acceptable to my readers to be presented with authentic copies of so much of them as relates to the present topic, together with a few practical remarks suggested by my own observation and experience; and, especially since his editor, to whom the one dated August 1808 was read, declared it to contain, in his opinion, by far the most complete explanation of the operation of any extant. I will beg only to add, that he kindly inclosed, with the first communication, two of his improved needles for my own immediate use.

"My dear Friend,

"I confide the method of operating which I pursue for cataract to your honor, and am very certain that it is safely deposited. I shall not have time to point out all the advantages which result from this deviation from the old method of couching, but, simple as they may appear, they are very important, as you will perceive, when I detail all the circumstances, which I shall sometime do in a Treatise on the Cataract.

"I always use the solution of belladonna*, and never begin the operation

* It ought to be generally understood, that the influence of the belladonna in producing a dilated condition of the pupil, is equally great when the extract, made of the consistence of cream, is thickly besmeared and suffered to remain for an hour or two over the skin of the palpebræ, as when it is introduced underneath them immediately upon the anterior surface of the globe of the eye. As I have repeatedly witnessed, in irritable and chronically inflamed states of the organ, a very considerable degree of excitement of the vessels of the conjunctiva, accompanied with severe temporary pain induced by the latter and more general mode of applying the remedy, I do not hesitate *exclusively* to recommend its *external* employment, which, whilst it alike secures the specific effect of the belladonna, is not productive of the slightest inconvenience in any respect. I cannot omit to add, that a strong infusion of the stramonium instilled into the eye, is also very effectual in causing an expansion of the pupillary aperture, with the advantage too of exciting infinitely less irritation and uneasiness than the former narcotic used in the same way. In a few cases I have known double and confused vision, and different kinds of ocular spectra, invariably follow its administration. Lady T. to whose eye I applied it at different times rather liberally, with the view of enlarging the pupil, which had become nearly obliterated from preceding internal ophthalmia, never failed to experience these effects to a most remarkable and alarming degree; nor did they ever entirely subside till some days had elapsed after the discontinuance of the belladonna. However, it must be admitted, that such is by no means the general consequence of its employment.

On operation until the pupil is as much dilated as it will admit of, keeping the eye, by means of Pellier's elevator, or else my own fingers, as steady as possible. The object of my introducing the instrument into the eye is, to cut the capsule in the anterior part of the crystalline; and therefore, as the lens is generally more dense towards the centre, I take care that it shall pass through the crystalline as near to the capsule as possible. That the instrument may traverse the lens freely, you will observe that it is made of the greatest admissible tenuity and flat, and that it cuts towards the point on each side. I find, by experience, that it can be conducted with care through the hardest lens; whereas the needles, such as Scarpa's and Hey's, only push the whole lens before them, and, without being able to carry the instrument to the capsule, the lens is made to press on and protrude the iris, *whence results the consequent inflammation*. As for the crystalline itself, you may or may not meddle with that: it may be well to loosen its texture in some instances, but you ought never to depress it. The instrument should enter the sclerotica

---

On the contrary, in by far the majority of instances, it seems to exert its peculiar agency solely upon the iris, the sympathetic as well as associated actions of which, for a time, it wholly destroys. The supposition that attributes this influence to its specific operation upon the radiated muscular fibres of the iris, is, however, unquestionably gratuitous; and many, at least, plausible arguments (which are fully enumerated in my lectures on the eye) might be adduced in opposition to that hypothesis. The iris possesses, indeed, in all its motions, a peculiar mode of action, differing essentially from what is observed in every other species of organised texture. Its usually expanded or passive condition in amaurosis proves that the contracted is a state of activity or violence; and its motions cease at the moment of death, and cannot, like those of muscular fibres, be renewed by the application of stimuli. And, when accidentally cut by falling before the knife in the operation of extraction, it hangs loosely pendulous in the aqueous humor, without manifesting any disposition to contraction. Blumenbach (and of a similar opinion was the illustrious Baron Haller) believes "the cause of its motions to depend on its vita propria or peculiar vital properties, since the iris," he remarks, "both in regard to its structure and vivid color, as well as with respect to its actions, is altogether singular and anomalous. Nor has he been able," he adds, "in a single instance, to detect the existence of muscular fibres, not even in the elephant and whale, any more than in the white rabbit." And, as we must believe in the identity of muscular fibres, and in their subserviency to the same laws in whatever situation they may be found, if the inspissated juice of the herb alluded to produces a dilatation of the pupil solely by virtue of its action upon the radiated muscular fibres of the iris, ought not, on that supposition, the orbicularis palpebrarum, with which the remedy is in much closer contact, to be at least simultaneously, if not primarily, affected?

 about about a line behind the ciliary ligament, and should be conducted through the anterior part of the crystalline, which is the softest. You may loosen the texture of the cataract before you divide the capsule or after, as in the operation seems most convenient, but the capsule must be divided at all events. I do not much care what becomes of the substance of the crystalline. I sometimes let it go in considerable quantity into the anterior chamber, if it seems tending that way, but I *never push* it, because that must press the iris.—N. B. Follow Hey's rule to be careful not to do too much.

"After the operation, the plan with me is purely antiphlogistic, and I believe you well know what that is. If your operation should not succeed at the first attempt, describe to me the appearances, and I will gladly give you my sentiments as to repeating it.

"With respect to congenital cataracts, from the repeated conversations we have had on the subject, it seems scarcely necessary for me to remind you that they are generally capsular, the whole or the greater part of the lens having probably been at some antecedent period, during the fœtal state, spontaneously absorbed. I shall only add to what I have already stated, that the steps to be pursued in the operation are nearly similar to those adopted for lenticular cataract, the great object being, either to make a sufficiently large central aperture for the rays of light to pass freely through it to the retina, or else to endeavor to tear the condensed capsule into as small fragments as possible, when it will become soluble in the aqueous humor, and be gradually absorbed; for which purpose you may use the needle with more freedom than in the former state.

With our united regards,

*Extracts from letters dated April and August*, 1808. I am, your's faithfully, J. C. SAUNDERS."

A more explicit, unaffected, and intelligible, relation of Mr. Saunders's method of proceedure in operating for cataract, could, I conceive, scarcely be penned, than what the above extracts from his valuable letters most satisfactorily exhibit. Nor can I suppose that any surgeon, at all conversant with ophthalmic practice, could fail, with the aid of such plain instructions, sufficiently to comprehend, and successfully to execute, the different steps of the above-described operation in common and favorable cases of cataract.

It may here be remarked, that, in the above statements, the *posterior* operation alone is detailed, the *anterior* having been adopted but a short time before that lamentable event took place, which deprived me of a sincere and esteemed friend; the public of one of its most philanthropic members, and the profession of a highly zealous and truly scientific practitioner.

Deducing, however, an opinion from my own individual experience, I do not think that the plan of introducing the needle through the cornea, instead of passing it in the usual manner manner behind the iris, is so great an improvement as might at first sight appear. For though, by that process, less pain and risque of subsequent inflammation are incurred, the more sensible and important parts of the organ thereby escaping injury, at the same time that the anterior lamella of the capsule of the lens is immediately exposed to the point of the needle; yet still the operator finds himself infinitely more restrained and embarrassed in the requisite movements of his instrument, and it is scarcely possible to act very freely and advantageously upon the cataract, without causing the evacuation of more or less of the aqueous humor; in which event the iris instantly contracts and advances towards the concave surface of the cornea; under which circumstance it certainly requires no small share of dexterity and caution to avoid doing mischief.

The first trial I made with the instruments sent to me by Mr. Saunders, (and which occurred very soon after their arrival,) was upon Hannah Dove, a poor woman, of a weakly constitution, and nearly forty years of age, from Sutton Ashfield, Nottinghamshire. She had labored under lenticular cataract in each eye for several preceding years, during which period she had borne four children, the features of none of whom had she ever had the happiness of beholding. She could, however, distinguish perfectly light from darkness, and, under favorable circumstances, was capable of decyphering the outlines of large objects. The pupils were circular, and sympathetically obedient to the different degrees of light admitted to the sensible retina. Agreeably to the above directions, I operated the same day upon both eyes in succession, assisted by my friend Dr. Marsden, senior physician of the General Hospital, near Nottingham. She described the pain as scarcely exceeding what would be inflicted by the prick of a pin.* The subsequent symptoms were

* The above instance, in respect to the trivial degree of pain produced by the operation, and the very mild inflammatory symptoms which supervened, may be regarded as illustrative of what has almost invariably occurred to the various patients upon whom I have successfully operated for the different species of cataracts, including a range of individuals from a very early to a very late period of life; my youngest patient having been only six months old, and the most advanced, Mrs. M. —— Hall, Norfolk, who has recently submitted to my operation on both eyes with every prospect of eventual restoration to sight, having attained her 84th year. Of the almost incredible ease to the patient with which the operation has been occasionally performed, Lieut. Col. F. is a remarkable example, were of the mildest description. On account of the hardness of the lens, I found great difficulty in reducing it to fragments, and it became requisite, in a few weeks, to repeat the process, which was not followed by any unfavorable symptoms. After this second operation she returned home; and, as absorption from that period was slowly progressive, and her vision gradually improving, it was not deemed expedient to introduce the needle a third time. The principal motive for my stating this case is, for the purpose of noticing the following curious and important pathological fact, namely, that, when the ruptured lens in each eye was scarcely more than half dissipated, she was attacked in so severe a manner by an epidemic ophthalmia as to threaten the immediate destruction of the organ; during the continuance of which, the absorptive process became altogether stationary. By the adoption of active antiphlogistic measures, the inflammatory symptoms were, however, eventually subdued; on the declension of which, the solution and absorption of the cataract were renewed with such astonishing vigor and rapidity, that in a very short period there was not a vestige of either left; the regenerated action of the absorbents having thus accomplished in a few days more than they had hitherto effected in as many preceding weeks. This event the husband announced to me in the following expressive words: "my wife's eyes are *quite clear now*," and added, "she can now see her family and friends, and manage her household affairs as formerly, which, from being so long blind, she never expected to do again." Nor had she, at the time he wrote, any assistance from spectacles, to obtain which was the chief object of his grateful letter.

Somewhat analogous to the above is the case of the Dowager Countess Spencer's female attendant, upon whose eye, at the desire of her ladyship, I operated last year for lenticular cataract. Although a highly diseased and debilitated subject, and far beyond her meridian, the operation

---

ample. Upon that gentlemen I operated some time ago, in the presence of two of our most eminent surgeons, for lenticular cataract in the right eye. The needle was introduced into the eye, the crystalline was broken, and the instrument again withdrawn, when in a minute or two after retiring from the sofa upon which my patient was placed for the purpose of undergoing the operation, to our mutual surprize and satisfaction, he entreated me not to keep him in a state of painful suspense; being absolutely unconscious that the process had been even commenced,—a fact that, at first, from the comparative exemption from pain with which it was executed, he could with difficulty be induced to believe.

excited excited very trifling inconvenience, and the process of solution and absorption of the lens, though slow, was going on quite equal to my most sanguine expectations, when, unfortunately, a very distressing intermittent humicrania occurred, and which, like the ophthalmia in the former instance, at once interrupted the further dispersion of the cataract. This accessory complaint was so violent as to resist the bark, arsenic, and various other remedies, for many successive weeks. At length, the paroxysms grew gradually milder, and finally terminated altogether upon a Sunday, at which time there existed so large a portion of the unabsorbed lens, as to render the sight exceedingly imperfect. She called upon me the following Tuesday, and informed me, with great joy, not only of the above event, but also of the restoration of her vision; being then capable of distinguishing objects as well as persons usually are who have lost the crystalline, the want of which being in some degree supplied by a proper convex glass, she could read the smallest print with perfect facility. From the closest examination I could not detect a particle of remaining cataract, the pupil was quite black and clear, and the iris completely circular, and actively alive to the different degrees of light transmitted to the retina.

Do not the above histories decidedly prove, that the absorptive process in the eye may, under certain circumstances, become in a great measure, if not entirely, suspended, in consequence of a new series of actions being established in the part, or its immediate neighbourhood, and on the cessation of which it may again revive with proportionably accumulated energy? Can the phenomenon depend upon the further secretion of aqueous humor becoming, under inflammatory action, wholly suppressed; in which case it is easy to conceive, that the quantity present at the time in the two chambers of the eye, having saturated itself with the disorganized lens, is thereby rendered incapable of effecting its further solutions? Without contending for the correctness of my hypothesis, the fact itself is unquestionable, and strongly inculcates the propriety and expediency of counteracting, by all practicable means, the occurrence of inflammation subsequent to the operation for cataract, as a circumstance that, independently of the danger to which it subjects the organ of vision, will infallibly arrest the rapid cure of the complaint. As far, however, as my own observations enable me to form a judgment on this point, I am inclined to believe, that such extraordinary instances of accelerated absorption are only to be anticipated in cases where the organ, perhaps functionally torpid, has been previously previously excited into a very unusual state of activity; and that, if the absorbents do not, on the *decline* of the inflammatory symptoms, *immediately* manifest such a disposition, we are not warranted in expecting that it will arise spontaneously hereafter. Accordingly, when the progress of absorption after the free disturbance of the crystalline, and laceration of its capsule, has seemed to flag and grow languid, I have endeavored to promote it by the application of powerful stimuli to the eye, and with very decided benefit, the cataract having in several instances, especially in children, disappeared so quickly in consequence, as to supercede the necessity of another operation, which, without such aid, I doubt not, would have been rendered indispensible. And, as an additional corroboration of the possibility of expediting the dissipation of the broken and disorganized lens, I will beg leave to subjoin the following interesting statement, which coincides with what Mr. Ware has already advanced upon the same subject.

A physician, some time since, brought for my inspection, rather as a matter of curiosity than from any expectation that the accident would admit of relief, a poor boy, about eleven years of age, who, having been just apprenticed to a shoemaker, and not having acquired the necessary dexterity in the use of his awl, inadvertently run it through the cornea of his right eye, and into the very substance of the lens. The necessary effect of the injury was the sudden formation of cataract, with the usual degree of accompanying blindness. The boy was forthwith conveyed to a general hospital, where measures calculated to arrest the progress of pain and inflammation, which speedily supervened, were very judiciously adopted. In the course of four or five weeks he was discharged, free from every symptom of irritation, but with scarcely any sense of sight. A fortnight afterwards he was brought to me, when I found the conjunctiva of its natural appearance, the cornea also perfectly transparent, except at the punctured part, where there existed a slight circumscribed opacity. The iris, though moveable, from having formed a partial adhesion to the capsule of the lens, was irregular in its pupillary margin. A very considerable portion of the lens hung projecting through the wound in the capsule into the anterior chamber of the eye. Having before met with a somewhat similar case, which terminated favorably, and being well aware of the powerful agency of the aqueous humor in promoting the solution of the lens when placed in free contact with it, I ventured to predict that the cataract would in all probability become spontaneously absorbed, and the boy in consequence recover his sight: a piece of information which excited in my friend no small share of surprise. However, I added, that the process might, I believed, be greatly accelerated by collateral aids. Requesting me to adopt any plan I thought would prove most conducive to that end, two active doses of calomel and jalap were prescribed to be given in the course of the ensuing week, and a lotion composed of the hydrarg. muriat. dissolved in equal portions of rose-water and camphor mixture with a small addition of æther, was also directed to be applied to the affected organ three times a-day. My patient called again after the expiration of a week, and reported to me, that the cathartic powders had acted with considerable violence, and that the collyrium had excited acute pain, which however subsided in a few minutes. With respect to the eye, I was pleased to observe that the projecting portion of lens had been wholly absorbed, and that there was an aperture formed in its centre of a sufficient size to admit of his seeing small objects through it very distinctly. By pursuing the same plan a week longer, the entire crystalline was dissolved and absorbed, and vision, in consequence, regained with a degree of perfection equal to what it ever is after the most successful operation for cataract.

The above instance affords, I think, very decisive evidence, that the absorbents of the eye may be excited into action by appropriate remedies, as well as those in other parts of the body, since, at the time those means, both general and topical, were had recourse to in the present case, the absorptive process had not even commenced, nor was there any substantial grounds for our expecting such a sudden event. Indeed, another case of a similar description that came under my observation soon afterwards, from a thorn having pierced the cornea and produced cataract, owing to the obstinacy of the parents in not allowing any measures to be adopted for its removal, remained stationary for several months; the result of which I have not had an opportunity of ascertaining.

(To be continued.)

## Figures and Tables

**Figure f1:**